# Psychological distress and resilience of mothers and fathers with respect to the neurobehavioral performance of small-for-gestational-age newborns

**DOI:** 10.1186/s12955-019-1119-8

**Published:** 2019-03-28

**Authors:** Mercedes Bellido-González, Humbelina Robles-Ortega, María José Castelar-Ríos, Miguel Ángel Díaz-López, José Luís Gallo-Vallejo, María Fernanda Moreno-Galdó, Macarena de los Santos-Roig

**Affiliations:** 1 Department of Developmental Psychology and Education, Faculty of Education Sciences, University of Granada, Granada, Spain; 2 Department of Personality, Evaluation and Psychological Treatment, Faculty of Psychology, University of Granada, Granada, Spain; 3 Gynecology Service, Virgen de las Nieves University Hospital, Granada, Spain; 4 Paediatrics Service, Virgen de las Nieves University Hospital, Granada, Spain; 5 Department of Methodology of Behavioral Sciences, Faculty of Psychology, University of Granada, Granada, Spain; 6 Department of Developmental Psychology and Education, Faculty of Education Sciences, University of Granada, Campus de Cartuja, 18071 Granada, Spain

**Keywords:** Parental psychological distress, Resilience, Pregnancy, Neurobehavioral performance, Small-for-gestational-age

## Abstract

**Background:**

The existence of psychological distress (PD) during pregnancy is well established. Nevertheless, few studies have analyzed the PD and resilience of mothers and fathers during high-risk pregnancy. This study analyzes the differences between parents’ PD and resilience and the relation between them and the neurobehavioral performance of their SGA newborns.

**Methods:**

This prospective study compares two groups of parents and newborns: case group (52 parents and 26 SGA fetuses) and comparison group (68 parents and 34 appropriate-for-gestational-age, AGA, fetuses). In each group, the parents were evaluated during the last trimester of pregnancy, to obtain standardized measures of depression, stress, anxiety, and resilience. At 40 ± 1 weeks corrected gestational age, psychologists evaluated the state of neonatal neuromaturity achieved.

**Results:**

Multivariate analysis of variance showed, in gender comparisons, that mothers obtained higher scores than fathers for psychological distress but lower ones for resilience. Similar differences were obtained in the comparison of parents’ distress to intrauterine growth by SGA vs. AGA newborns. Mothers of SGA newborns were more distressed than the other groups. However, there were no differences between the fathers of SGA vs. AGA newborns. Regarding neurobehavioral performance, the profiles of SGA newborns reflected a lower degree of maturity than those of AGA newborns. Hierarchical regression analyses showed that high stress and low resilience among mothers partially predict low neurobehavioral performance in SGA newborns.

**Conclusions:**

These findings indicate that mothers of SGA newborns may need psychological support to relieve stress and improve their resilience. Furthermore, attention should be paid to the neurobehavioral performance of their babies in case early attention is needed.

## Background

The mother’s wellbeing is the primary condition for the proper organization of child development, from the moment of conception. However, this wellbeing may be disrupted by pregnancy-related concerns [1], such as discovering that the baby is small for gestational age (SGA). These newborns can be detected during pregnancy by fetal biometry, and fetal weight can be calculated by ultrasound examination. Thus, from a very early stage, we can monitor the development of a population representing 5–10% of live births [2] in which there is a significant possibility of a disability developing.

Medical supervision of this population is performed according to a strict protocol [3] which calls for periodic obstetric reviews and, among other procedures, a detailed ultrasound scan in each such review.

Parents can suffer psychological distress (PD) both from receiving the bad news of inadequate fetal growth, due to the possible consequences for the baby’s future development [4], and from being present during an ultrasound scan, due to preoccupation about the baby’s progress [5].

PD is determined by the level of stress perceived and by emotional manifestations of a depressive and/or anxious nature, in response to the adjustments required of persons faced with stressful experiences [6, 7].

Various adverse effects of PD during pregnancy on fetal development have been identified [8, 9], such as the risk of premature birth or of low birth weight [8, 10–12]. In such cases, cognitive, behavioral, and emotional problems may later arise [13–15].

However, when pregnant women experience chronic stress, and their babies, therefore, are at risk of adverse development, they are more likely to be able to cope if they have high levels of resilience [1]. Accordingly, we believe it of interest to study resilience as a dynamic, multidimensional construct, defined as the ability to successfully withstand a threatening, challenging situation, to recover from a situation of extreme distress and/or trauma or even to prosper in the midst of adversity [16]. Resilience does not imply invulnerability to stress, but rather the ability to recover from negative events [17, 18]. Thus, persons who are resilient are capable of mobilizing resources and of successfully adapting to severe adversity [19]. Resilience, therefore, can be viewed as an index of mental health [20].

In relation to pregnancy or complications arising during this period, some studies have observed that high levels of resilience can be a protective variable, as this quality is associated with low levels of depression and with a better quality of life, both in mothers diagnosed with preeclampsia [21] and in those at risk of premature birth [22]. However, few researchers have examined the role of resilience in parents when SGA fetus is diagnosed.

Moreover, previous research has tended to ignore the impact of this situation on the father, although recent studies have highlighted the existence of differences between women and their partners in terms of PD, reporting that mothers tend to suffer higher levels of depression and anxiety [23]. These differences increase as the pregnancy progresses, and are greatest in the final trimester [24]. A recent review on paternal depression suggested that programs should be established to detect and evaluate PD in both parents [25].

Among populations at risk of developing symptoms of PD – for example, the parents of preterm infants – anxiety and depression levels exceeding risk thresholds have been found, affecting mothers to a greater extent than fathers [26].

In the population of SGA newborns and their parents analyzed in this study, previous research has not established whether emotional wellbeing and emotional health are similar in mothers and fathers. Also lacking are data on the relation between the emotional states of each parent, their degree of resilience and the neurobehavioral performance of the SGA baby. The present study addresses these gaps in the literature.

Our initial hypothesis is that the mothers and fathers of SGA newborns will present higher levels of PD and less resilience than parents of AGA newborns, and that this has implications for the newborns’ neurobehavioral performance.

## Methods

This preliminary study was prospective, with inter-group comparison (AGA/SGA), and conducted as a prior step to undertaking a research project focused on determining the effectiveness of a program of psychological attention to enhance the emotional health of parents and their ability to provide stimulation to the fetus and to promote the health and development of their child – in short, to support parenting skills during the first year of life of the SGA infant (Trial Registration: ISRCTN 15627704).

### Participants

Participants were selected from the 897 pregnant women, together with their partners and their live born newborns, who were treated at the Virgen de las Nieves Hospital (Granada, Spain) during the last quarter of 2015.

### Inclusion criteria

The case group was composed of mothers and fathers and their SGA newborns. During the study period, approximately 5% (45 newborns) were SGA and this diagnosis remained unchanged during the successive ultrasound examinations performed throughout the third trimester of the pregnancy. SGA was defined as fetal weight below the 10th percentile, in accordance with the guidelines on Management of the Small-for-Gestational-Age Fetus published by the RCOG [3].

The comparison group was formed of mothers, fathers and their AGA newborns (fetal weight > 10th percentile). The selection of AGA newborns for analysis was performed in the same period as that for the SGA newborns (the day after the diagnosis), applying similar comparison criteria regarding gender and maternal education (primary school, secondary school, and university/college education).

### Exclusion criteria

The following were excluded from the study population: the parents of fetuses presenting hypoxic ischemic encephalopathy (HIE) (1 case); parents who were drug users, presented a psychiatric disorder or were currently receiving psychological treatment (1 case); parents whose mother tongue was not Spanish (2 cases); and parents who did not provide informed consent (11 cases). In 4 cases, the parents lived in other cities, which prevented them from participating in the evaluation of their SGA newborns. These cases, too, were excluded from the study group.

Finally, the case group consisted of 52 mothers and fathers and 26 SGA fetuses. In every case, the birth weight of the newborns was below the 10th percentile. Of these, 24 newborns were discharged from hospital without incident, with their mothers, while two remained in the intensive care unit.

The comparison group consisted of 68 mothers and fathers and 34 AGA fetuses, selected from among the AGA fetuses evaluated by ultrasound during the last trimester of pregnancy, the day following the detection of each SGA baby recruited to the case group and matched by the parents’ education level (primary school, high school, or college/university) and sex of the fetus. The parents thus selected were included in the study on provision of informed signed consent to participate.

### Measures

#### Biomedical parameters

Protocolized ultrasound monitoring [3, 27] was performed by a specialist obstetrician for all the pregnant women in our study groups, with AGA and SGA fetuses. The routine ultrasound examinations were performed in accordance with the criteria established for SGA pregnancies by the Royal College of Obstetricians and Gynaecologists [3]. According to these criteria, multiple ultrasound examinations are required when SGA is diagnosed. This approach reveals whether the situation persists until the end of pregnancy, since the scan at 37 weeks increases the detection rate of SGA [28]. In addition, each baby’s neonatal status was observed for four consecutive hours by a specialist pediatrician, taking into account the Queensland Maternity and Neonatal Clinical Guideline on SGA babies [29, 30]. No obvious neurological abnormalities were apparent in any baby.

#### Psychological evaluation

Neonatal behavior and maternal and paternal PD and resilience were assessed by the psychologists, who had previously trained in the application of the study tests. The pyschological data were collected during the third trimester of pregnancy, which is when mothers present the highest levels of stress [24].

The following instruments were employed.

- *Edinburgh Postnatal Depression Scale (EPDS)* [31, 32]. This 10-item scale assesses the subject’s mood during the previous seven days. The response options range from 0 “always or most of the time” to 3 “never”. The Spanish version of this scale provides good validity, sensitivity and specificity [32]. In our sample, the Cronbach’s α index score was 0.82. We also calculated the test-retest correlation with a small sample to check the stability of the scores at two months, with R_test-retest_ = 0.60.
- *Pregnancy-Related Anxiety Scale (PRAS)* [33]. The Spanish version of the PRAS was used to assess the subjects’ anxiety/fear related to pregnancy and childbirth. This scale was administered both to the fathers and the mothers, adapting the ten questions as appropriate. The questions focused on the last month, with a response scale ranging from 1 “not at all/never” to 4 “a great deal/almost always”. The version used by Rini et al. (1999) presented indices of reliability (Cronbach’s α) of 0.78 for the English-language version and 0.80 for the Spanish version. The corresponding indices of reliability for our sample were α = 0.81 and R_test-retest_ = 0.56.
- *Perceived Stress Scale (PSS)* [34], Spanish-language version [35]. This scale measures the extent to which life situations are considered to be stressful. The Spanish version, consisting of 14 items related to the previous month, offers a range of responses from 0 “never” to 4 “very often” and presents acceptable indices of reliability (α = 0.81 and R_test-retest_ = 0.73) and good evidence of concurrent validity and sensitivity [34]. The corresponding indices of reliability for our sample were α = 0.85 and R_test-retest_ = 0.64.
- *Resilience Scale (CD-RISC)* [36]. This scale, too, was used to assess the ability of the parents, in each group, to cope with adversity. The scale consists of 10 items scored on a 5-point Likert scale ranging from “0 = never” to “4 = almost always”. The items included in this instrument address personal characteristics such as self-efficacy, flexibility, emotional self-control, strength and sense of humor. The scale offers high reliability (α = 0.85, R_test-retest_ = 0.71) and validity [37]. In our study sample, the indices of reliability were α = 0.85 and R_test-retest_ = 0.45.
- *The Neonatal Behavioral Assessment Scale* (NBAS, 4th edition) [38]. The purpose of this scale is to assess the full range of behavioral responses of the newborn (aged 0–2 months) within an interactive context, composed of the child and the examiner. The scale consists of two types of tests or items: behavioral and reflex responses. The items are grouped into seven clusters: habituation (HAB) (the ability to respond to and inhibit discrete stimuli while asleep); orientation (ORI) (the quality of overall alertness and the ability to respond to visual and auditory stimuli); motor (MOT) (motor performance and the quality of movement and tone); range of states (RANS) (arousal and lability); regulation of states (REGS) (the baby’s ability to regulate his/her state in response to increasing levels of stimulation); autonomic stability (AUTS) (signs of stress related to homeostatic adjustments of the central nervous system); and reflexes. In addition, supplementary items can be used to evaluate signs of fragility or vulnerability. In this study, the supplementary item included was the cost of attention (ATEN), which measures the extent to which the engine and physiological system are stressed. The NBAS items are quantified on a 9-point scale, where 9 = best execution, except in eight cases where best execution is represented by the central score of 5. A psychometric evaluation of the NBAS scale, applied to a sample of Spanish children, obtained a mean reliability of 0.78 by Cronbach’s α [39]. In our study sample, the alpha score, by sub-scales, ranged from 0.70 (AUTS) to 0.94 (ORI).

### Procedure

All procedures performed in studies involving human participants were in accordance with the ethical standards of the institutional and/or national research committee and with the 1964 Helsinki Declaration and its later amendments or comparable ethical standards. This study has been approved by Ethical Research Committee of the Virgen de las Nieves Hospital, Granada, Spain (date: September 14, 2015, registration number: 0864-N-15). Informed consent was obtained from all individual participants included in the study.

The parents and their newborns were evaluated and monitored by the medical team (gynecologists and a pediatrician) and by psychologists from the Virgen de las Nieves third-level hospital in Granada (Spain). The gynecologists’ evaluation was carried out by ultrasound examination of the gestation process and of fetal development in utero. Four measurements were obtained: biparietal diameter (BPD), head circumference (HC), abdominal circumference (AC) and femur length (FL), and these were used to approximate the fetal weight. If SGA was diagnosed, the volume of amniotic fluid was determined, a fetal-placental Doppler study was performed and, by serial ultrasound monitoring, intrauterine growth (IG) was evaluated. All these actions were taken with the express consent of the woman concerned and her partner.

The psychologists conducted extensive interviews with the mothers and fathers, requesting their informed consent to participate in the study, and seeking information about their lifestyle and family background. Data were obtained on the parents’ emotional state, in both groups, via questionnaires focused on any stress, depression and anxiety experienced and on the parents’ resilience, presented in a counterbalanced order. The evaluation was conducted in a quiet, separate room, offering adequate privacy. The same instructions were given in all cases.

At 40 ± 1 weeks of corrected gestational age, psychologists – blinded to the study group and perinatal outcomes – evaluated neonatal neuromaturation, according to the NBAS. The newborns were evaluated between feeds, in a room within the hospital that was small, quiet and dimly lit, with a temperature between 22 °C and 27 °C, in the presence of the mother and if possible, of the father, too [38]. All evaluations were performed by one of three observers accredited by the Brazelton Institute (Harvard Medical School, Boston, USA).

A complete and detailed evaluation of the newborn was performed by the pediatrician during the first 48 h after birth [29].

### Statistical analysis

Before the analyses, nonparametric (χ^2^, Mann-Whitney) and parametric (Student t) tests were performed to confirm the homogeneity of the comparison groups. After verifying the initial equivalence of the groups as regards the sociodemographic variables, a multivariate analysis of variance (MANOVA) was carried out with gender (father/mother) and intrauterine growth, IG, (AGA/SGA) as factors. The aim of this analysis was to determine the simple effects and interactions of these factors on PD and resilience. Once the presence of interactions was confirmed, post hoc Bonferroni tests were run to identify statistically significant differences between the four groups (mothers AGA/SGA and fathers AGA/SGA) concerning PD and resilience.

Finally, regression analyses were performed to analyze the predictive ability of IG and PD or resilience on the newborns’ outcomes (neurobehavioral development). Taking into account that PD and resilience are continuous variables, various hierarchical multiple regressions were performed, using the PROCESS macro for SPSS (Model 1) for fathers and mothers [40]. IG, PD and resilience were introduced as predictor variables. The IG condition was coded as − 0.5 for AGA and 0.5 for SGA newborns. The continuous predictor variables (PD or resilience) were centered before computing the interaction terms [41]. The criterion variables were all the components of the NBAS Scale. The simple effects and the interaction of the predictor variables (IG and PD, or resilience) on the criterion variables were then analyzed.

## Results

The tests performed showed that there were no significant differences in any of the sociodemographic and clinical characteristics considered (Table 1). Thus, the groups were homogeneous regarding sociodemographic variables. However, differences were observed in the clinical variables concerning gestational age, birth weight and the Apgar scores at one minute (apgar1) and at five minutes (apgar5), which were significantly higher in the AGA group than in the SGA group (*p* < 0.05).

**Table 1:**
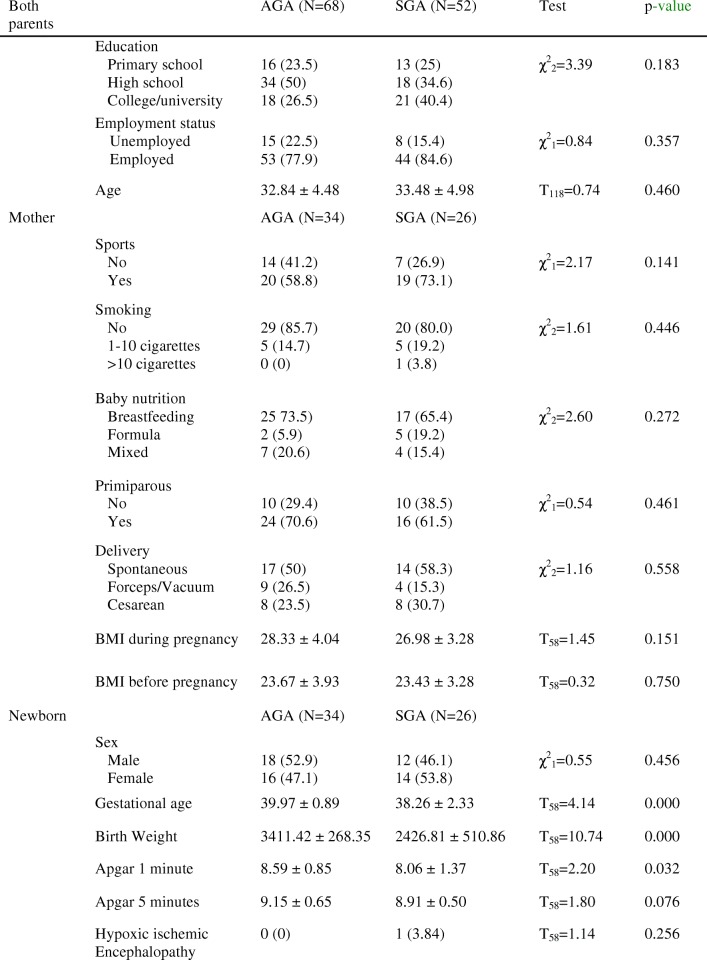
Sociodemographic data and clinical variables in the comparison groups

Note. Data are given as n (%), mean ± SD. *p*-values were calculated using Student’s t-test, or Pearson’s chi-square test

In the case group, two newborns remained in the NICU, and the remaining 24 were discharged. Although these two cases represented only 7% of the total sample of SGA infants, we examined whether their neurobehavioral development was significantly different from that of the other SGA infants (not NICU), before testing the study hypotheses. To do so, we applied the procedure described by Crawford et al. [42, 43], for comparing a case (NICU-SGA infant) with a small sample of comparison cases (non NICU-SGA infants). This test revealed no significant differences in neurobehavioral development, either in the first case (AUTS, t(24) = 0.81; MOT, t(24) = − 0.18; HAB, t(24) = − 0.13; RANS, t(24) = 1.26; REGS, t(24) = 0.28; ORI, t(24) = − 0.88; ATEN, t(24) = − 1.14, all with non-significant *p*-values) or in the second (AUTS, t(24) = − 0.20; MOT, t(24) = − 1.38; HAB, t(24) = −0.12; RANS, t(24) = 0.30; REGS, t(24) = − 0.74; ORI, t(24) = − 0.87; ATEN, t(24) = − 1.12, all with non-significant *p*-values).

In relation to PD, the mothers of these NICU-SGA newborns did not differ from the other mothers of SGA infants (Mother 1: EPDS, t(23) = 1.54; PSS, t(22) = no data available; PRAS, t(23) = − 0.91; CD-RISC, t(23) = 1.74, all with non-significant *p*-values; Mother 2: EPDS, t(23) = 0.04; PSS, t(22) = − 0.12; PRAS, t(23) = − 0.29; CD-RISC, t(23) = − 0.65, all with non-significant p-values. Likewise, no differences were found among the fathers of the infants (Father 1: EPDS, t(18) = 1.88; PSS, t(16) = no data available; PRAS, t(16) = 0.50; CD-RISC, t(16) = − 1.78, all with non-significant *p*-values; Father 2: EPDS, t(18) = − 1.04; PSS, t(16) = -0.26; PRAS, t(16) = − 0.27; CD-RISC, t(16) = − 2.87 all with non-significant p-values except for CD-RISC in which case *p* < 0.05).

### MANOVA results for psychological distress and resilience

In the MANOVA, the factors included were gender (father/mother) and intrauterine growth (AGA/SGA). The results shown in Table 2 reflect the main effects produced by gender on all the variables analyzed for depression, perceived stress, anxiety and resilience. The mothers obtained significantly higher scores than the fathers for PD, and lower ones for resilience. A similar pattern was observed for intrauterine growth. The SGA newborns’ parents presented significantly higher scores for PD and lower ones for resilience than those of AGA newborns, although anxiety was only marginally significant.

**Table 2:**
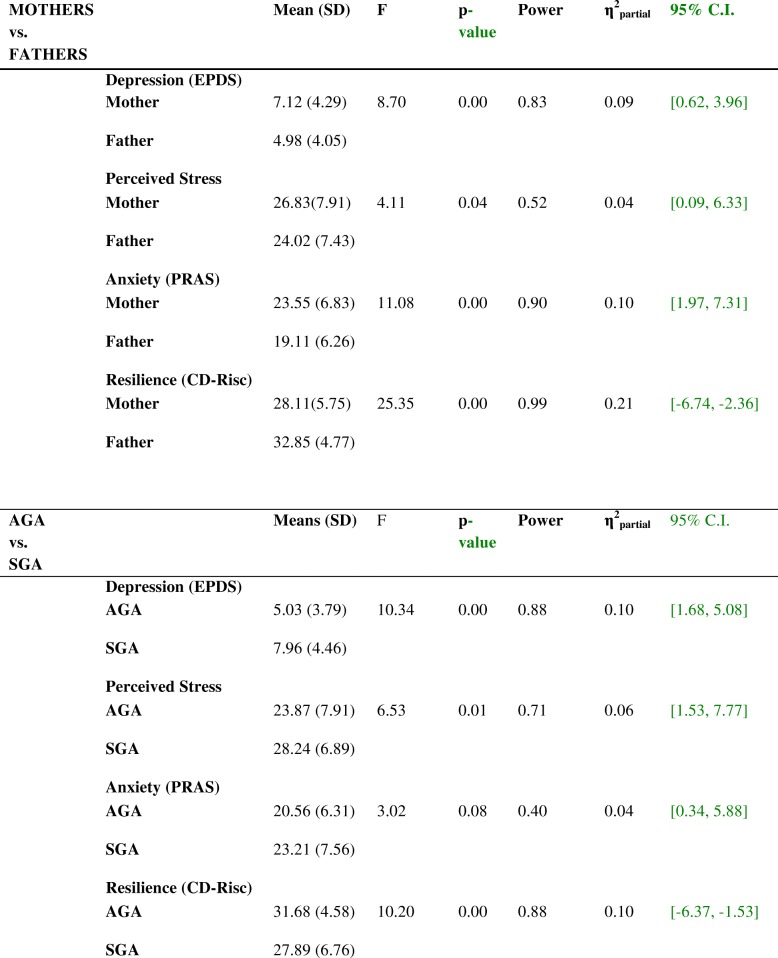
MANOVA results for differences by *gender* (mothers vs. fathers) and by *intrauterine growth* (AGA vs. SGA) in depression, perceived stress, anxiety and resilience

AGA = Appropriate for Gestational Age, SGA = Small for Gestational Age

With respect to the interaction between gender (father/mother) and intrauterine growth (AGA/SGA), Table 3 shows that the results among the four groups were significant for depression (*p* = 0.05) and for resilience (*p* = 0.01).

**Table 3:**
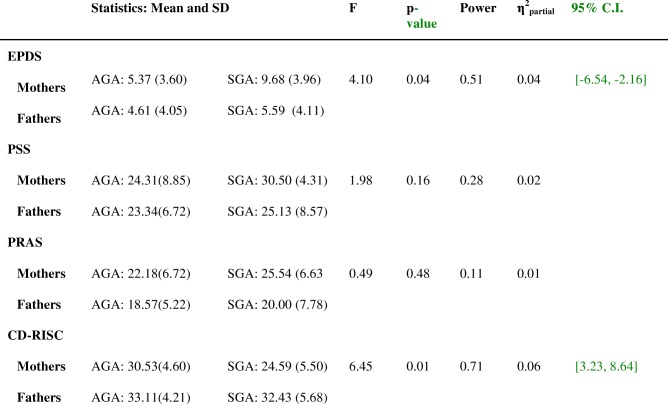
MANOVA results for the interaction between gender and intrauterine growth in depression, perceived stress, anxiety and resilience

EPDS = Edinburgh Postnatal Depression Scale, PSS=Perceived Stress Scale, PRAS=Pregnancy-Related Anxiety Scale, CD-RISC = Resilience Scale, AGA = Appropriate for Gestational Age, SGA = Small for Gestational Age

The mothers of SGA newborns presented higher values for depression (p = 0.05) and lower ones for resilience (*p* = 0.01) than the other three groups (the mothers and fathers of AGA newborns and the fathers of SGA newborns) (Table 3). This was confirmed by post hoc Bonferroni tests. The mothers of SGA newborns had significantly higher scores for depression (t = 3.25, *p* = 0.00) less resilience than the fathers in the same group (t = − 4.87, *p* = 0.00) and also had poorer results in depression and resilience than the mothers of AGA newborns (t = 4.13, *p* = 0.00 and t = − 4.36 p = 0.00), respectively). The fathers of SGA and AGA newborns did not differ with respect to any variable.

### Relation between intrauterine growth condition and the child’s neurobehavioral performance: the moderation of psychological distress and resilience

In general, IG had a significant effect on the newborns’ neurobehavioral performance. The SGA newborns scored significantly lower than the AGA ones in all neurobehavioral dimensions: autonomic stability, t(58) = 2.24, *p* = 0.02 (95% CI = [−.14, 1.15]), motor performance, t(58) = 7.68, *p* = 0.00 (95% CI = [− 0.09, 0.55]), habituation, t(58) = 7.10, *p* = 0.00 (95% CI = [0.06, 1.15]), range of states, t(58) = 2.19, *p* = 0.03 (95% CI = [− 0.04, 1.04]), regulation of states, t(58) = 7.08, *p* = 0.00 (95% CI = [1.50, 2.69]), orientation, t(58) = 11.52, *p* = 0.00 (95% CI = [2.21, 3.15]), and cost of attention, t(58) = 11.30, *p* = 0.00 (95% CI = [2.74, 3.93]).

### The moderation of mothers’ psychological distress and resilience

Regarding the simple effects of the mothers’ PD and resilience on their newborns’ performance, the results were partially significant. Depression scores were not predictors of any dimensions of the newborns’ performance. Maternal stress had a marginally significant effect on NBAS autonomic stability (NBAS-AUTS), and resilience was only a statistically significant predictor of NBAS-AUTS (Table 4). However, analysis of the interactions showed that IG and mothers’ stress strongly interacted with NBAS-AUTS and NBAS-MOTOR (Table 4). For high levels of maternal stress, IG had a statistically significant effect on NBAS-AUTS, t = − 3.01, *p* = 0.00 (95% CI = [− 2.10, − 0.42]) and NBAS-MOT, t = − 7.09, *p* = 0.00 (95% CI = [− 2.50, − 1.40]). The SGA newborns in this group presented the poorest levels of performance.

**Table 4:**
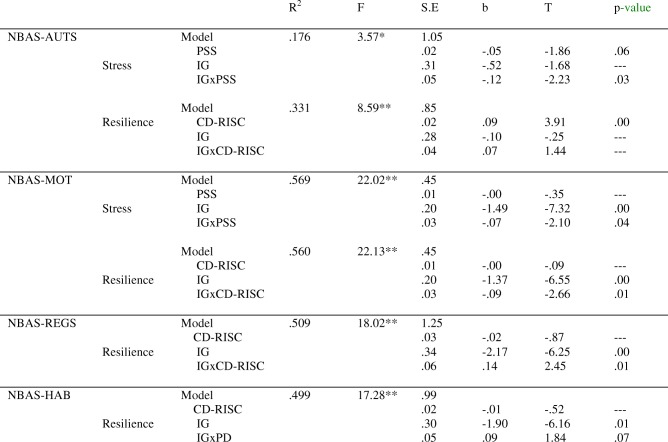
Neurobehavioral outcomes (AUTS, MOT, REGS, HAB) as a function of intrauterine growth and mothers’ stress and resilience

Note. *p < .05, **p < .01

NBAS Neonatal Behavioral Assessment Scale, AUTS autonomic stability, MOT motor, RGES regulation of states, HAB habituation

The interaction between IG and mothers’ resilience is a predictor of NBAS-MOT, NBAS-REGS, and (with marginal significance) NBAS-HAB (Table 4). Although the results show that SGA newborns perform less well than AGA newborns, the difference is smaller when mothers present high scores for resilience. Thus, NBAS-MOT, t = − 2.69, *p* = 0.00 (95% CI = [− 1.41, − 0.20]) and NBAS-REGS t = − 2.63, *p* = 0.01 (95% CI = [− 2.32, − 0.31]) in the newborns whose mothers have high levels of resilience, while for those with less resilience NBAS-MOT, t = − 6.64, *p* = 0.00 (95% CI = [− 2.51, − 1.34]) and NBAS-REGS t = − 6.28, p = 0.00 (95% CI = [− 4.00, − 2.06]).

### The moderation of fathers’ psychological distress and resilience

For the fathers, depression (EPDS) is a marginally significant predictor of NBAS-AUTS, b = 0.07(.03), t(44) = 1.93, *p* = 0.06. Paternal anxiety, both alone and in interaction with IG, has a statistically significant effect on newborns’ NBAS-REGS (Table 5). Fathers’ high anxiety during pregnancy deteriorates the regulation states of AGA newborns, t = − 3.83, *p* = 0.00 (95% CI = [− 2.63, − 0.81]).

**Table 5:**
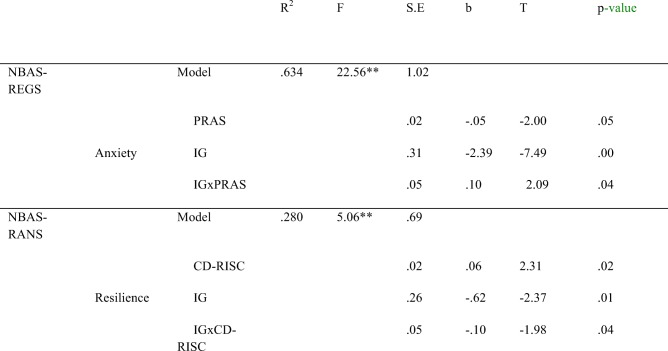
Neurobehavioral outcomes (REGS, RANS) as a function of intrauterine growth and fathers’ anxiety and resilience

Note. m.s. marginally significant, *p < .05, **p < .01

NBAS Neonatal Behavioral Assessment Scale, RGES regulation of states, RANS range of states

The resilience of fathers (CD-RISC) is a statistically significant predictor of NBAS-RANS. Together, IG and resilience produced a significant interaction effect on newborns’ NBAS-RANS (Table 5). Thus, for high levels of paternal resilience, statistically significant differences were observed in the AGA newborns, who obtained the highest scores, t = − 3.06, p = 0.00 (95% CI = [− 1.88, − 0.38]).

## Discussion

In this preliminary study, we assess the PD experienced by mothers and fathers of SGA children, their ability to overcome PD related to this circumstance (i.e., their resilience) and the influence played by this adaptive process on the neurodevelopment of the SGA newborn.

In general, both mothers and fathers experience pregnancy and its circumstances with anticipation and a certain level of stress, because the mere fact of an ultrasound examination to determine the evolution of the fetus can provoke anxiety [5], although possibly at differing levels between the two parents. Our findings show that symptoms of PD are stronger in mothers than in fathers and that most of the significant differences observed had a medium-high effect size (η^2^ partial). This finding is consistent with previous studies conducted across diverse samples of parents with healthy pregnancy [23, 24]. According to Redshaw and Henderson [44], these differences can be interpreted as reflecting less concern and engagement among the fathers, who do not share the strong emotions experienced by mothers.

Similarly, the mental health of parents exposed to a stressor such as the concern caused by the SGA status of the newborn is poorer than that of persons not exposed to this factor. In other words, the parents of fetuses diagnosed SGA presented more symptoms of depression, anxiety and stress than the parents of AGA fetuses. In addition, resilience levels were lower in the SGA group. Our results are based on the parents’ responses to questionnaires on PD and resilience, but they are consistent with those based on physiological responses to stress [10, 45, 46]. Consequently, these findings confirm and support each other.

It should be noted that prior studies in this field did not take into account that the fact of preoccupation about the risks posed by anomalous fetal growth is itself a stressor. Other factors that have not been examined previously include the mental health of the parents, its interaction with intrauterine growth and the effects this may have on the child. To fill this research gap, we examined the interaction between the gender variable (mothers, fathers) and intrauterine growth (SGA, AGA). Our analysis showed that the mothers of SGA newborns experience higher levels of depression and lower levels of resilience. These findings may indicate that the father’s level of engagement with his newborn becomes apparent somewhat later [45].

Another aspect distinguishing our study from previous research is that we analyzed resilience. Interestingly, while the mothers of SGA newborns were less resilient than those of AGA newborns, there was considerable similarity between the fathers. This suggests that, in general, fathers and mothers present different responses related to pregnancy trimester [24], which would confirm the importance of gender roles in emotional health during pregnancy [47]. In line with Cock et al. [48], we believe that reducing stress and promoting resilience in the father could have a protective effect on the mother and would thus be beneficial for the child’s subsequent development. The present preliminary study should be extended with a longitudinal one, to determine the protective effect of the father’s mental health on the SGA baby, since the paternal presence, in itself, has been shown to influence the outcome of preterm and low birth weight deliveries [49] .

In our study, less mature profiles of neurological behavior were observed among SGA than AGA newborns, especially as concerns habituation, orientation, motor, range of states, regulation of states, autonomic stability and cost of attention. In this respect, our results are consistent with those of Padidela and Bhat [50], who also reported differences in all the NBAS domains, and partially so with Feldman and Eidelman [51], who only observed differences in the orientation and motor domains. In addition, we recorded low levels of resilience and high levels of PD in the mothers of SGA newborns, which could partially account for the poor neurobehavioral performance observed [52, 53].

Hierarchical regression analyses were performed to determine the relation between IG (SGA/AGA) and PD or resilience. The results obtained show that high stress in the mother partially predicts some neurobehavioral outcomes of SGA newborns. Thus, stress explains between 17.6 and 56.9% of the variance of behavioral responses of the newborn, such as signs of stress, motor performance, quality of movement and tone. Similar results were obtained for resilience, which explains between 33.1 and 56% of the variance of behavioral responses, including in addition to the two aspects mentioned above, the newborn’s ability to respond to and inhibit discrete stimuli while asleep and to regulate states in response to increasing levels of stimulation. These results confirm our initial hypothesis about the negative consequences of high PD and low resilience among mothers for SGA babies’ neurobehavioral performance.

However, the situation is different for fathers, among whom high levels of anxiety and low ones of resilience affect (also partially) AGA, but not SGA newborns. We interpret these results as meaning that fathers are less involved during the initial stages [45] or that they tend to adopt a protective attitude in the face of adversity that allows them to control their emotional state [54, 55]. In any case, it is clear that the primary caregivers present a common neural basis for maternal and paternal care [56] and therefore that responses to pregnancy and parenting will be similar if both situations are jointly addressed by the primary caregivers.

Our study has certain limitations. First, it is difficult to make causal statements about the impact of PD and resilience on the neurodevelopment of the newborn. On the one hand, the roles of maternal and paternal cognitive abilities were not evaluated, and this might contribute both directly and indirectly to neonatal neurodevelopment. However, beyond the impact of shared genetics, prenatal stress can induce programming effects on the neurocognitive development and behavior of the newborn [46, 57].

Second, the parents’ subsequent responses to parenthood may differ from those shown during the pregnancy. Therefore, in future research it would be interesting to conduct a longitudinal follow-up to determine the evolution of the couple’s emotional state in relation to motherhood, fatherhood and parenting. Nevertheless, the newborn stage (the first 28 days of life) is of crucial importance in childcare, and PD can be aggravated during this time, at the beginning of motherhood and fatherhood, following the recent experience of a risky pregnancy and of childbirth, and in relation to early intervention to ensure the infant’s proper development. Analysis of the discussions in focus groups has shown that during the first weeks after childbirth, mothers experience a greater psychological burden and often report symptoms of postpartum depression [58]. For all these reasons, we believe that the period considered in this study merits specific attention.

Another possible weakness of this research is that we did not evaluate the mental health of mothers and fathers in the first and second trimesters of pregnancy. However, a reliable prediction of SGA is not usually made until the third trimester [59]. Moreover, according to the literature this final period of pregnancy is the target period for PD and its consequences, regarding the possibility of a SGA newborn being born [8].

Despite these weaknesses, our study has various strengths. First, in the study design: the psychologists taking part were also expert evaluators of infant development, and this fact decreases the possibility of reporter bias that might otherwise lead to a spurious association being deduced. Second, maternal and paternal PD were evaluated with diverse instruments, which decreases the possibility of misclassification of outcomes [60]. Finally, good power and effect size (partial η^2^) were obtained in the analyses [61, 62].

## Conclusions

Our findings suggest that mothers do not show the same level of emotional health during pregnancy as their male partners, according to the symptoms of PD presented. In addition, levels of resilience are lower among mothers than fathers. Both of these circumstances are more strongly apparent in mothers of fetuses diagnosed SGA. Neurobehavioral performance among SGA newborns is immature with respect to that shown by AGA newborns. This outcome might be exacerbated by high stress and low resilience among the mothers; both factors can predict neurobehavioral performance in the newborn. Finally, we believe that in future research, psychological intervention programs should be developed, especially in mothers of SGA newborns, seeking to reduce PD, to increase resilience and to promote the sharing of parenting responsibilities during pregnancy and the newborn’s early life.

## Abbreviations

AGA: Appropriate-for-Gestational-Age
ATEN: Attention
AUTS: Autonomic stability
CD-RISC: Resilience Scale
EPDS: Edinburgh Postnatal Depression Scale
HAB: Habituation
HIE: Hypoxic ischemic encephalopathy
MOT: Motor
NBAS: The Neonatal Behavioral Assessment Scale
ORI: Orientation
PD: Psychological Distress
PRAS: Pregnancy-Related Anxiety Scale
PSS: Perceived Stress Scale
RANS: Range of states
RCOG: Royal College of Obstetricians and Gynaecologists
REGS: Regulation of states
SGA: Small-for-Gestational-Age
